# After scaling to body size hip strength of the residual limb exceeds that of the intact limb among unilateral lower limb prosthesis users

**DOI:** 10.1186/s12984-023-01166-z

**Published:** 2023-04-25

**Authors:** Andrew Sawers, Stefania Fatone

**Affiliations:** 1 Department of Kinesiology, University of Illinois at Chicago, 1919 West Taylor Street, Rm. 646, Chicago, IL 60612 USA; 2 Department of Physical Medicine and Rehabilitation, Northwestern University, Chicago, IL 60611 USA; 3 Department of Rehabilitation Medicine, University of Washington, Seattle, WA 98195 USA

**Keywords:** Amputation, Amputee, Muscle strength, Rehabilitation

## Abstract

**Background:**

Hip muscles play a prominent role in compensating for the loss of ankle and/or knee muscle function after lower limb amputation. Despite contributions to walking and balance, there is no consensus regarding hip strength deficits in lower limb prosthesis (LLP) users. Identifying patterns of hip muscle weakness in LLP users may increase the specificity of physical therapy interventions (i.e., which muscle group(s) to target), and expedite the search for modifiable factors associated with deficits in hip muscle function among LLP users. The purpose of this study was to test whether hip strength, estimated by maximum voluntary isometric peak torque, differed between the residual and intact limbs of LLP users, and age- and gender-matched controls.

**Methods:**

Twenty-eight LLP users (14 transtibial, 14 transfemoral, 7 dysvascular, 13.5 years since amputation), and 28 age- and gender-matched controls participated in a cross-sectional study. Maximum voluntary isometric hip extension, flexion, abduction, and adduction torque were measured with a motorized dynamometer. Participants completed 15 five-second trials with 10-s rest between trials. Peak isometric hip torque was normalized to body mass × thigh length. A 2-way mixed-ANOVA with a between-subject factor of leg (intact, residual, control) and a within-subject factor of muscle group (extensors, flexors, abductors, adductors) tested for differences in strength among combinations of leg and muscle group (α = 0.05). Multiple comparisons were adjusted using Tukey’s Honest-Difference.

**Results:**

A significant 2-way interaction between leg and muscle group indicated normalized peak torque differed among combinations of muscle group and leg (p < 0.001). A significant simple main effect of leg (p = 0.001) indicated peak torque differed between two or more legs per muscle group. Post-hoc comparisons revealed hip extensor, flexor, and abductor peak torque was not significantly different between the residual and control legs (p ≥ 0.067) but torques in both legs were significantly greater than in the intact leg (p < 0.001). Peak hip abductor torque was significantly greater in the control and residual legs than the intact leg (p < 0.001), and significantly greater in the residual than control leg (p < 0.001).

**Conclusions:**

Our results suggest that it is the intact, rather than the residual limb, that is weaker. These findings may be due to methodological choices (e.g., normalization), or biomechanical demands placed on residual limb hip muscles. Further research is warranted to both confirm, expand upon, and elucidate possible mechanisms for the present findings; and clarify contributions of intact and residual limb hip muscles to walking and balance in LLP users.

***Clinical Trial Registration*:**

N/A.

**Supplementary Information:**

The online version contains supplementary material available at 10.1186/s12984-023-01166-z.

## Background

Hip muscles play a prominent role in the biomechanical adaptation to unilateral lower limb amputation [1, 2]. Unilateral lower limb prosthesis (LLP) users compensate for the loss of ankle and/or knee muscle function by recruiting ipsilateral hip muscles to produce propulsive, stabilizing, and body weight supporting forces during locomotor activities [1–5]. Hip muscles in transfemoral prosthesis users may also serve to stabilize their residual limb within the socket [6, 7] and provide a degree of control over the prosthesis [3]. Given their expansive set of responsibilities, it is perhaps not surprising that residual and intact limb hip muscle weakness [8–13] has been associated with a host of gait impairments including reduced walking speed [5, 8, 10, 11, 14], increased metabolic cost [15–17], decreased balance confidence [18], abnormal joint loading [8, 14], as well as reduced mobility [19] and walking endurance [20, 21]. Hip strength may therefore prove to be an appealing target for interventions that seek to improve walking and balance performance in LLP users.

There is currently no consensus regarding the extent of hip strength deficits in unilateral LLP users [22]. In the absence of agreement, suitable targets for rehabilitation cannot be clearly identified, impeding the development, testing, and implementation of specific, evidence-based physical therapy interventions. Identifying patterns of hip muscle weakness may also expedite research seeking to identify mechanisms of muscle dysfunction in LLP users. To date, many [5, 12, 14, 21, 23], but not all [10, 11] studies involving transtibial prosthesis users report *no* significant difference in hip strength between the residual and intact limbs, regardless of hip muscle group. Studies of transfemoral prosthesis users typically report residual limb hip muscles as significantly weaker than their intact limb counterparts, but the specifics (i.e., which muscles) varies from study to study [8, 9, 13, 24]. To advance our understanding of hip strength deficits in unilateral LLP users several historically overlooked factors must be addressed [22]. First, hip strength must be interpreted in the absence of the confounding effects of age, gender, and body size [25, 26]. Age- and gender-matched controls can be recruited to address the former [9, 12, 27], while the biological influence of body size (i.e., muscle mass) on muscle strength can be addressed by normalizing strength data to appropriate anthropometric variable(s) [28], ensuring unbiased comparisons between people and legs that differ in size [26, 29, 30]. Second, documentation of hip strength across all four major hip muscle groups, in both the residual and intact limbs, is required to characterize within and between limb patterns of hip strength among unilateral LLP users [11, 13, 19, 31]. Finally, nearly half of the evidence concerning hip strength in unilateral LLP users is based on data collected almost 20 years ago [22]. Changes in amputation technique and immediate post-operative care, a decline in the provision of rehabilitation services, an aging and increasingly co-morbid population, as well as advances in prosthetic design may affect hip strength in LLP users and our understanding of it, necessitating the collection of further data.

The purpose of this study was to test whether hip extension, flexion, abduction, and adduction muscle strength, estimated by maximum voluntary isometric peak torque, and normalized to body mass x thigh length, differed between the residual and intact limbs of unilateral LLP users, as well as age- and gender-matched controls. Based on previous literature [8–11, 13, 24] and structural changes to residual limb hip muscles [32–35], we hypothesized that the residual limb would be the weakest of the three legs, regardless of hip muscle group. We also hypothesized that given the transection of several hip muscles during transfemoral amputation, hip strength would be significantly lower in transfemoral versus transtibial prosthesis users.

## Methods

### Study design

A cross-sectional study was conducted to determine the effect of amputation level (i.e., transfemoral and transtibial), leg (i.e., residual, intact, and control), as well as muscle group (i.e., extensors, flexors, abductors, and adductors) on hip strength, as estimated by maximum voluntary isometric peak torque, in established unilateral lower limb prosthesis (LLP) users, as well as age- and sex-matched controls. Study protocols were reviewed and approved by an institutional review board at the University of Illinois at Chicago. All individuals provided written informed consent prior to participation.

### Participant recruitment

Individuals with a unilateral transtibial and transfemoral amputation due to trauma, dysvascular complications, cancer, or infection were recruited from prosthetic clinics in Chicago using convenience sampling. To participate, LLP users were required to be 18 years of age or older; have a history of wearing a prosthesis for at least two years post amputation; be able to walk short distances (e.g., 10 m); and be able to read, write, and speak English. LLP users were excluded if they had a congenital amputation, a second amputation, contralateral complications, or a neuromusculoskeletal or cardiopulmonary condition that would preclude them from completing testing procedures. Individuals without amputation were recruited from the community as controls using convenience sampling. Controls were matched to individual LLP users based on gender and age ± 5 years [27].

### Data collection

#### Participant characterization

Participant age, gender, and amputation characteristics (e.g., etiology, time since amputation) were collected via self-report, while the Medicare Functional Classification Level (MFCL) (i.e., K-level) [36] of LLP user participants was determined via interview by a certified prosthetist. The perceived physical function and fatigue of LLP users and controls were assessed by administering the PROMIS-29 Physical Function and Fatigue scales [37, 38], respectively. Perceived physical function specific to LLP users was documented by administering the Prosthetic Limb Users Survey of Mobility (PLUS-M) [39]. The number of co-morbidities was characterized by administering the Charlson Comorbidity Index (CCI) [40]. Body mass, height, and thigh length (ASIS to medial femoral condyle or distal end of residual limb) were also recorded to aid in the normalization of peak hip torque.

#### Hip torque data collection

Maximum voluntary isometric hip extension, flexion, abduction, and adduction torques were measured using a motor-driven dynamometer (Biodex System 4 Pro, Biodex Medical Systems, Inc., Shirley, NY) [41]. When testing hip extension or flexion, participants were placed in a supine position [8, 42] with the hip flexed to 20 degrees [5]. To test abduction or adduction, participants assumed a side-lying position [12, 14, 42, 43], with the hip abducted 10 degrees [5, 10, 42]. Testing order (i.e., leg and muscle group) was randomized, and the prosthesis was removed when testing the residual limb [8, 9]. Following three-submaximal practice trials [44], participants performed 15 five-second maximum voluntary effort isometric trials with 10 s of rest between trials. Instructions to participants were to generate maximum voluntary isometric force as quickly as possible, and to hold that maximum effort until told to relax. The analog signal from the dynamometer was sampled at 1000 Hz, beginning just prior to the verbal “go” command. Verbal encouragement was provided during the 5-s contraction. Five-minute rest periods were implemented between the testing of each muscle group.

### Data processing and analysis

#### Hip torque data processing

The maximum voluntary isometric peak torque for each muscle group in each leg was derived from the digitized analog signal (NI USB-6341, National Instruments, Austin, TX) after adjusting for the effects of gravity, and smoothed using a low-pass Savitzky-Golay filter. Isometric peak torque was selected owing to the simplicity of its performance, prevalence in previous LLP user research [22], consideration as a purer test of muscle strength [24], and correlation with other more complex metrics of muscle function (e.g., average power, impulse) [45, 46] and muscle action (e.g., isotonic, and isokinetic) [47]. Peak torque was calculated as the maximum torque recorded between signal onset and offset across all 15 trials. Data processing steps were run using custom MATLAB (MathWorks, Natick, MA) routines. Mathematically adjusting for the biological influence of body size on muscle strength is necessary to create measures of hip torque that are independent of confounding anthropometric variables, and suitable for comparison between people and legs that differ in size [26, 29, 30]. Based on prior research [28], peak hip torque was normalized to body mass x thigh length (BM × TL) using allometric scaling [25, 26, 48–50]. Based on the principle of geometric similarity [25], non-normalized hip torque (S) was modeled as a power function S = S_n_ (BM × TL)^β^, where (S_n_) is normalized hip torque, and (β) is the scaling exponent [25, 26, 51, 52]. To determine appropriate values for the scaling exponent of each muscle group and leg combination, the power function was log transformed, and standard linear regression was used to calculate the slope of the resulting linearized equation, log (S) = log (S_n_) + β (log BM x TL) [48]. Peak torque values for each muscle group and leg combination were then scaled to BM × TL by inserting the corresponding β-value into the re-written power function, S_n_ = (S)/(BM × TL)^β^. Normalization of peak torque values was conducted using SPSS v.28 (Chicago, IL).

#### Statistical analysis

Departures from normality among continuous variables were evaluated with Shapiro–Wilk tests [53]. Peak hip torque values, normalized to body mass × thigh length were identified as outliers and removed if they exceeded a threshold of ± 2.5 median absolute deviations (MAD) above or below the median [54]. Measures of central tendency and dispersion, or frequency and proportion, were calculated to describe continuous and categorical characteristics of the study sample, respectively. Independent-samples t-tests, or Mann–Whitney U tests, were run to test for differences in characteristics (e.g., age, perceived physical function) between LLP users and matched controls.

Using only the data of LLP users, a three-way mixed ANOVA with one between-subject factor of amputation level and two-within-subject factors of leg and muscle group was run to determine whether the effects of leg and muscle group on maximum voluntary isometric peak torque were dependent on amputation level. The absence of a significant 3-way interaction between amputation level, leg, and muscle group would indicate that the effects of leg and muscle group on peak hip torque were *not* dependent on amputation level. Similarly, the absence of significant 2-way interactions between muscle group and amputation level, or leg and amputation level, would indicate that peak torque values did not differ according to combinations of muscle group and amputation level, or leg and amputation level, respectively. Transtibial and transfemoral prosthesis users could subsequently be combined into a single group of LLP users for analysis with respect to matched controls.

A two-way mixed ANOVA with a between-subject factor of leg (3-levels: intact, residual, control), and a within-subject factor of muscle group (4-levels: extensors, flexors, abductors, adductors), was run to test for differences in peak isometric hip torque among combinations of leg and muscle group. Assumptions of homogeneity of variances and covariances, as well as sphericity in the dependent variable (i.e., normalized peak torque) were evaluated with Levene’s test of homogeneity, Box’s test of equality, and Mauchly’s test of sphericity, respectively. The level of significance for all tests was set to α ≤ 0.05. Multiple comparisons during post-hoc tests were adjusted using Tukey’s Honest Significant Difference (HSD) test. All statistical analyses were performed with SPSS v.28 (Chicago, IL).

## Results

### Participant characteristics

Twenty-eight unilateral lower limb prosthesis (LLP) users, 14 transfemoral and 14 transtibial, as well as 28 age- and gender-matched controls participated in the study (Table 1). The cause of amputation was non-dysvascular in 21 (75%) of the LLP users, and dysvascular in seven (25%). Fifty percent had a K3 Medicare Functional Classification Level (K2: n = 14, K3: n = 14,), and the median time since amputation, which was non-normally distributed (W = 0.857, p = 0.001) was 12 years with an interquartile range of 17 years. All the transfemoral prosthesis users wore microprocessor knees, and 23 of the 28 participants had non-articulating energy storage and return feet, with the remaining five participants using a multiaxial foot. LLP users’ PLUS-M T-scores (median: 51.7, IQR: 7.47) were non-normally distributed (W = 0.879, p = 0.004). The number of co-morbidities, PROMIS-29 Physical Function T-scores, and PROMIS-29 Fatigue T-scores were non-normally distributed (LLP users: W ≤ 0.889, p ≤ 0.009; controls: W ≤ 0.835, p < 0.001), while age, body mass, and height were normally distributed (LLP: W ≥ 0.950, p ≥ 0.231; controls: W ≥ 0.928, p ≥ 0.068). Mann–Whitney U tests revealed no statistically significant differences between LLP users and matched controls in age, body mass, or height, (U ≥ 250.5, z ≥ − 1.60, p ≥ 0.109) (Table 1). Perceived physical function (i.e., PROMIS-29 Physical Function T-scores) was significantly lower in LLP users than matched controls (U = 544, z = 4.07, p ≤ 0.001) (Table 1), while the number of co-morbidities and perceived fatigue (i.e., PROMIS-29 Fatigue T-scores) were significantly greater in LLP users than matched controls (U ≤ 232, z ≤ − 2.21, p ≤ 0.027) (Table 1). Median thigh length was non-normally distributed (W ≤ 0.917, p ≤ 0.03) and not significantly different (U = 250, z = − 1.60, p = 0.109) between the intact leg of LLP users (0.43 m) and that of controls (0.42 m). Median residual limb thigh length among transfemoral prosthesis users (0.26 m) was normally distributed (W = 0.921, p = 0.260) and significantly shorter than that of transtibial prosthesis users (0.42 m) (U = 0.12, z = − 4.52, p < 0.001).

**Table 1:** Demographic, health, and mobility-related characteristics common to lower limb prosthesis (LLP) users as well as age- and gender-matched controls (CONT)

	Group	Median (Q1, Q3)	p-value
Age (years)	LLP	55.0 (44.0, 60.8)	0.993
	CONT	55.0 (39.5, 62.8)	
Body mass (kg)	LLP	82.2 (68.7, 100.2)	0.641
	CONT	78.3 (64.3, 96.1)	
Height (m)	LLP	1.74 (1.68, 1.82)	0.133
	CONT	1.71 (1.65, 1.78)	
PROMIS-29 Physical function	LLP	41.8 (37.9, 48.3)	< 0.001
	CONT	57.0 (57.0, 57.0)	
PROMIS-29 Fatigue	LLP	48.6 (46.0, 55.1)	0.003
	CONT	43.1 (33.7, 48.6)	
CCI	LLP	1 (0, 2)	0.027
	CONT	0 (0,1)	

*CCI* Charlson Co-morbidity Index; *PLUS-M* Prosthetic Limb Users Survey of Mobility; *Q1* first quartile; *Q3* third quartile

### Peak hip extension, flexion, abduction, and adduction torque

#### Need for and effectiveness of normalizing peak hip torque

In all four hip muscle groups, across all three limbs, peak isometric torque was significantly associated with body mass × thigh length (BM × TL), indicating that normalization was required for valid and fair comparisons between people and legs that differ in size. Associations between peak torque and BM × TL were *non-linear* in the residual and control limbs, as well as the hip extensors of the intact limb. In the remaining intact limb hip muscle groups, peak torque had a *linear* association with BM × TL (Supplemental Material 1). Normalization successfully removed the association between peak torque and BM × TL in all four muscle groups, across all three limbs, producing body size independent measures of hip torque suitable for comparison between participants and legs that differed in size (Additional file 1).

#### Identification and adjustment for statistical assumptions with a mixed ANOVA

Peak torque values normalized to BM x TL exceeded the outlier threshold of ± 2.5 median absolute deviations [54] in one transfemoral and one transtibial prosthesis user. Both LLP users, and his/her matched control, were therefore excluded from further analyses. Normalized peak torque (Table 2) was then log-transformed so that values approximated a normal distribution for any combination of amputation level, leg, and hip muscle group (W ≥ 0.868, p ≥ 0.050). Homogeneity of variance and covariance of the normalized and log-transformed peak hip torque values were confirmed by Levene’s test of equality of variance, p ≥ 0.298, and Box’s test of equality of covariance, p = 0.116, respectively. Mauchly’s test of sphericity revealed that the assumption of sphericity was violated for the three-way interaction between amputation level, leg, and muscle group, X^2^(5) = 14.40, p = 0.013. Greenhouse–Geisser corrections were therefore applied to the interpretation of the mixed-ANOVA output.

**Table 2:** Normalized peak isometric torque (% BM × TL) for residual and intact limb hip muscle groups in unilateral lower limb prosthesis users, as well as age- and gender-matched controls. Data are presented as median ± median absolute deviation (MAD)

Residual limb	Intact limb	Control limb
Peak isometric hip extension torque		
25.7 ± 7.35	8.52 ± 2.03	22.4 ± 5.14
Peak isometric hip flexion torque		
17.3 ± 5.81	2.44 ± 0.882	18.1 ± 6.04
Peak isometric hip abduction torque		
26.5 ± 7.71	2.15 ± 0.667	21.9 ± 5.48
Peak isometric hip adduction torque		
13.5 ± 4.75	2.06 ± 0.638	8.40 ± 1.59

*BM* body mass; *MAD* median absolute deviation; *TL* thigh length

#### The effect of amputation level on hip muscle strength: 3-way mixed ANOVA

After applying a Greenhouse–Geisser correction for the violation of sphericity, the three-way interaction between amputation level, leg, and muscle group on normalized and log-transformed peak torque in LLP users was not statistically significant, F(1.44, 34.6) = 1.29, p = 0.279. The absence of a significant three-way interaction indicates that the interpretation of any two-way interaction between amputation level, leg, or muscle group (e.g., leg × muscle group) on normalized and log-transformed peak torque was not dependent on the third remaining factor (e.g., level of amputation). Similarly, 2-way interactions between muscle group and amputation level, F(1.40, 33.7) = 0.059, p = 0.885), as well as leg and amputation level, F(1, 24) = 0.001, p = 0.885), were not statistically significant. The absence of significant two-way interactions indicates that normalized and log-transformed peak torque values did not differ according to combinations of muscle group and amputation level, or leg and amputation level. These results indicate that the effects of leg and muscle group on normalized maximum voluntary isometric peak torque were *not* dependent on amputation level. Transtibial and transfemoral prosthesis users were therefore combined into a single group of LLP users in all subsequent analyses.

#### The effect of leg and muscle group on hip strength: 2-way mixed ANOVA

There was a statistically significant two-way interaction between leg and muscle group on normalized and log-transformed peak torque values, F(5.17, 194) = 78.8, p < 0.001. The significant two-way interaction between leg and muscle group indicates that normalized and log-transformed peak torque values differed according to combinations of muscle group (e.g., hip extensors, abductors) and leg (i.e., residual, intact, or control leg). Consequently, simple main effects of leg on each muscle group (i.e., between leg differences), and muscle group on each leg (i.e., within leg differences) were tested and interpreted using univariate and repeated measures ANOVA procedures, respectively. Pairwise comparisons were performed for all significant simple main effects.

#### Between leg comparisons: simple main effects of leg on hip muscle group and accompanying pairwise comparisons

**Fig. 1:**
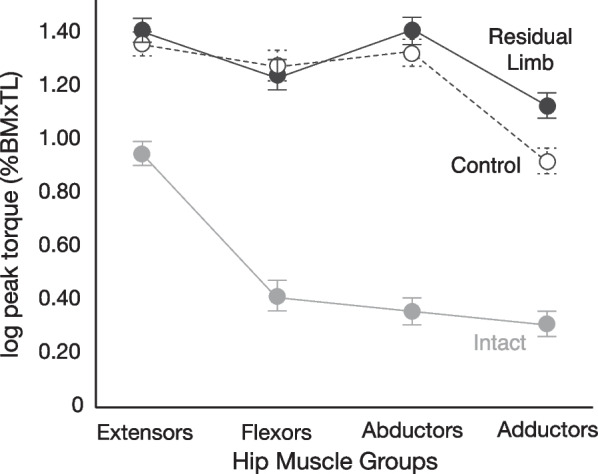
Within and between limb differences in log transformed, isometric peak torque values (mean ± 95% CI) normalized to body mass × thigh length for the hip extensor, flexor, abductor, and adductor muscle groups in the residual (filled black, solid line) and intact (filled grey, solid line) limbs of unilateral lower limb prosthesis users, as well as age- and gender-matched controls (filled white, dashed line). Isometric peak torque was significantly greater (i.e., p < 0.001) among all four hip muscle groups in the residual and control limbs when compared to the intact limb. Except for hip adduction, peak torque values were not significantly different between the residual and control legs (p ≥ 0.067)

All torque data are reported as mean % BM x TL ± 95% CI. Simple main effects of leg on hip muscle group were considered statistically significant at a Bonferroni-adjusted alpha level of 0.0125 (i.e., 4 simple main effects, one per muscle group). There was a statistically significant simple main effect of leg on peak torque for each hip muscle group, F(2, 75) ≥ 130.6, p < 0.001, indicating that normalized and log-transformed peak torque differed between two or more legs for each hip muscle group. Post-hoc pairwise comparisons, examined with a Tukey HSD adjusted p-value of 0.0167, (i.e., comparisons between three legs), revealed that normalized and log-transformed peak torque values for the hip extensor, flexor, and abductor muscle groups were not significantly different between the residual and control legs (p ≥ 0.067) (Table 3; Fig. 1). However, values from the residual and controls legs were significantly greater than those in the intact leg (p < 0.001) (Table 3, Fig. 1). Peak hip adduction torque was also significantly greater in the control and residual legs compared to the intact leg (p < 0.001) (Table 3, Fig. 1), yet unlike the other hip muscle groups, peak hip adduction torque was significantly greater in the residual than the control leg (p < 0.001) (Table 3, Fig. 1).

**Table 3:** Log-transformed peak isometric hip torque normalized to body mass × thigh length in the residual and intact limbs of unilateral lower limb prosthesis users as well as age- and gender-matched controls. Data are presented as mean ± 95% confidence interval

	Hip extensors (HE)	Hip flexors (HF)	Hip abductors (ABD)	Hip adductors (ADD)	Simple main effects of muscle group
Residual limb (RL)	1.40 ± 0.10	1.23 ± 0.14	1.40 ± 0.12	1.12 ± 0.11	(HE = ABD) > (HF > ADD)^b^
Intact limb (INT)	0.927 ± 0.11	0.401 ± 0.12	0.350 ± 0.10	0.303 ± 0.11	HE > (ABD = HF = ADD)^b^
Control (CONT)	1.35 ± 0.07	1.27 ± 0.08	1.32 ± 0.09	0.916 ± 0.07	(HE = ABD = HF) > ADD^b^
Simple main effects of leg	(RL = CONT) > INT^a^	(RL = CONT) > INT^a^	(RL = CONT) > INT^a^	(RL > CONT) > INT^a^	

^a^Pairwise between leg comparisons, p ≤ 0.0167

^b^Pairwise within leg comparisons, p ≤ 0.0083

#### Within leg comparisons: simple main effects of hip muscle group within each leg and accompanying pairwise comparisons

All torque data are reported as mean % BM × TL ± 95% CI. Simple main effects of hip muscle group were considered statistically significant at a Bonferroni-adjusted alpha level of 0.0167 (i.e., 3 simple main effects, one per leg). There was a significant simple main effect of muscle group on normalized and log-transformed peak torque within the residual leg, F(2.14, 53.6) = 69.3, p < 0.001, intact leg, F(2.51, 62.8) = 247.1, p < 0.001, and control leg, F(2.60, 64.9) = 189.6, p < 0.001, indicating that normalized and log-transformed peak torque differed between two or more hip muscle groups within each leg. Post-hoc pairwise comparisons, examined with Bonferroni adjusted p-values (i.e., 0.0083, six comparisons between four muscle groups), revealed that within the residual limb, normalized and log-transformed peak torque was not significantly different between the hip extensors and abductors (p = 0.98), but both were significantly greater than the flexors or adductors (p < 0.001) (Table 3, Fig. 1). Normalized peak hip flexion torque was also significantly greater than peak hip adductor torque (p = 0.007) (Table 3, Fig. 1). Within the intact leg, normalized and log-transformed peak torque was significantly greater in the hip extensors than the flexors, abductors, and adductors (p < 0.001) (Table 3, Fig. 1). Peak torque was not significantly different however, between the flexors, abductors, or adductors (p ≥ 0.018) (Table 3, Fig. 1). Within the control leg, normalized and log-transformed peak torque was not significantly different between the hip extensors, flexors, and abductors (p ≥ 0.041), but all three were significantly greater than peak torque in the adductors (p ≤ 0.001) (Table 3, Fig. 1).

## Discussion

The objective of this study was to test whether hip muscle strength, estimated by maximum voluntary isometric peak torque, and normalized to BM x TL, differed between the residual and intact limbs of unilateral LLP users, as well as age- and gender-matched controls. In contrast to previous research [5, 9, 12, 14, 21], and our own hypothesis, the results suggest that it is the intact, rather than the residual limb, that is the weakest of the three legs. Direct comparisons across the literature are however limited by the substantial methodological variation across studies. Notable variations in data collection and analysis throughout the literature include testing posture (e.g., supine, sitting, or standing) and joint angle [5, 21, 23], testing equipment (i.e., computerized versus handheld dynamometer) [11–13, 19, 20], mode of muscle action (i.e., isometric versus isokinetic) [5, 8, 11, 13, 23], whether the prosthesis is worn [10–12, 21, 23] or removed [5, 8, 9, 13, 19, 24, 28, 31] while testing the residual limb, gravity compensation, familiarization (i.e., number of trials) [55], and normalization for confounding anthropometric variables [22]. The adoption of standardized methods for data collection, processing, and reporting of strength-related outcomes in LLP users would enable the comparison and aggregation of data across studies. Below we describe how elevated and prolonged activation of residual limb hip muscles during ambulatory activities may act to preserve or restore residual limb hip muscle strength in the face of reduced physical activity. Next, we explain how normalization, a key methodological choice, may reveal otherwise obscured between limb differences in hip strength among unilateral LLP users. Finally, we highlight clinical implications of the results, and proposed future research needs.

### Elevated and prolonged activation of residual limb hip muscles while walking may offset reduced physical activity, preserving, or restoring residual limb hip strength in unilateral lower limb prosthesis users

Physical activity among LLP users is characterized by limited volume [56–59], duration [56, 58], and intensity [57, 60]. For example, LLP users take between 1540 and 4000 steps per day [57–64], well below physical activity guidelines for the general population (i.e., 10,000 steps per day) [65, 66] or adults with a disability or chronic illness (i.e., 5500 to 6500 steps per day) [67]. While lower body muscle strength would be expected to decrease with reduced physical activity and the accompanying disuse of lower limb muscles [9], residual limb hip muscles may be less susceptible to the adverse effects of inactivity than intact limb hip muscles. Specifically, residual limb hip muscles remain active over a longer period of the gait cycle [3, 4, 6, 68–70], than their intact limb counterparts or the same muscles in age- and gender-matched controls [6, 70, 71]. Whether meant to compensate for the loss of ipsilateral ankle and/or knee muscle function [1–4, 72–74], stabilize the residual limb within the socket [6, 7], adapt to advances in powered prosthetic technology [75], or provide control over the prosthesis [3], the elevated and prolonged activation of residual limb hip muscles during each step [3, 4, 6, 68–70] may have the unintended benefit of offering a degree of protection against the weakening effects of reduced physical activity that drives intact limb hip muscle weakness. The prolonged activation of residual limb hip muscles may therefore preserve, or with time, restore residual limb hip muscle strength in unilateral LLP users by increasing their “use” per step. Beyond the additional research required to investigate the association between physical (in)activity, hip muscle activation, and hip muscle strength in unilateral LLP users, efforts to identify other potential mechanistic explanations for the between and within limb differences in hip strength observed in the current study are required.

### Detection of between limb differences in hip strength among unilateral LLP users may depend on identifying and adjusting for confounding anthropometric variables

While historically considered to be weaker [8, 9, 24], hip muscles in the residual limb of unilateral LLP users were found to be as strong or stronger than those in the intact limb, or those of age- and gender-matched controls (Table 3, Fig. 1). Unlike much of the research conducted to quantify hip strength in LLP users to date [22], here, peak torque was scaled to BM × TL, with the aim of mathematically adjusting for the biological influence of body size on muscle strength [28]. While limited, the use of allometric scaling has been shown to alter the interpretation of strength data in LLP users [28], and other clinical populations [29, 48, 76]. Here, allometric scaling, and in particular the values of the scaling exponents (i.e., β) used to adjust for *linear* and *non-linear* associations observed between non-normalized peak torque and BM x TL, may have revealed otherwise obscured between limb differences in hip strength. Among controls and the residual limb of LLP users, non-normalized peak torque (S) had a *non-linear* association with BM x TL (Supplemental Material 1). The resulting scaling exponents therefore assume smaller values (i.e., between zero and 1.0) [48] than those used to adjust for the *linear* associations between non-normalized peak torque and BM × TL in the intact limb of LLP users (i.e., β = 1) [48] (Supplemental Material 1). When applied to the re-written power function, S_n_ = (S)/(BM × TL)^β^, the smaller *non-linear* scaling exponents (β) have the effect of reducing the size of the denominator and, in turn, increasing the magnitude of normalized peak torque values (S_n_) relative to those of the intact limb in LLP users (Table 2). Identifying and adjusting for linear and non-linear associations between peak torque and confounding anthropometric variables appear therefore to have a considerable influence on the interpretation of hip strength data among unilateral LLP users. Consequently, between limb differences in hip muscle strength among LLP users may be revealed only when appropriately scaled to body size. Given the apparent importance of normalization to the interpretation of hip muscle function in unilateral LLP users, additional research is required to identify and establish biomechanically-sound, clinically feasible, and standardized approaches to the normalization of muscle function in unilateral LLP users [28].

### Several important considerations for the assessment and rehabilitation of unilateral LLP users emerge from the observed within and between limb hip strength differences

Gait deviations [10, 12, 13], reduced walking speed and endurance [5, 11, 14, 20, 21, 77, 78], as well as increased metabolic cost [16] have historically been associated with weakness in the *residual limb* of unilateral LLP users. Several recent studies have however reported that *intact limb* muscle function may also play a substantial role in determining walking endurance [79] and physical activity levels [19] among unilateral LLP users. The results of these latter studies, and our discovery that once scaled to body size the intact not residual limb hip muscles appear weaker, injects uncertainty into whether walking and balance performance in unilateral LLP users is limited primarily by intact or residual limb muscle function. Additional research using body size independent measures of muscle function is required to clarify the contributions of intact and residual limb hip muscles to walking and balance performance in unilateral LLP users [22, 80]. Rehabilitation protocols that focus on strengthening intact limb muscles as much or more than those in the residual limb may also be warranted.

The strength of the residual limb hip muscles in the current study suggests that determining how residual limb hip muscle torques can be most efficiently transferred through the prosthesis to the ground may have important implications for walking and balance performance. Controlled experimental conditions were used to isolate and quantify the torque generating capacity of residual and intact limb hip muscle groups. Whether this torque generating capacity generalizes to functional activities, whereby the “strongest” LLP users also possess the ability to generate the greatest hip torques while walking with their prosthesis, and do so in an efficient manner, remains unknown. Similarly, factors that mediate the efficiency with which residual limb hip muscle torques contribute to propulsive, braking, stabilizing, and body-weight supporting forces while walking remains unknown. Prosthetic-specific factors including socket designs, interfaces, and alignment; biomechanical factors such as co-contraction; rehabilitative factors like gait training; and physiological factors such as pain, may all contribute to the efficiency with which unilateral LLP users are able to generalize residual limb hip strength to walking and balance performance. The advancement of prosthetic technology (e.g., powered ankles and knees), as well as the use of assistive devices (e.g., a cane), may also alter the demands placed on residual limb hip muscles, influencing their strength, and potentially the efficiency with which they contribute to key locomotor requirements (e.g., propulsion). Assistive technology, including hip exoskeletons, may also have strength-related applications among LLP users, both to supplement and to strength weak hip muscles [81]. Our results would indicate that such applications may be applicable to the intact as well as the residual limb. Identification of modifiable factors, be they physiologic, prosthetic, or rehabilitative, which maximize the efficiency of force transmission from residual limb hip muscles through the prosthesis may enhance walking and balance performance in unilateral LLP users.

Existing clinical tests of lower body muscle function would be unable to identify either the between or within limb strength deficits described in the current study. Contemporary, standardized clinical tests of lower body muscle function are largely based on variations of timed sit-to-stand tasks [82–85]. Compared to computerized dynamometers, the five-times sit-to-stand test and the 30 s sit-to-stand test engage multiple muscle groups across and within the intact and residual limb of unilateral LLP users [83, 86], often in unique, varied, and asymmetric patterns [87, 88]. Such compensations, coupled with the inability to evaluate individual muscle groups, may mask important muscle- and limb-specific strength deficits, limiting the ability of clinicians to provide personalized treatment. Consequently, while existing clinical tests of lower body muscle function may provide a generic assessment of how strong or weak a LLP user is, they cannot specify where weakness resides, limiting the ability of clinicians to intervene. Existing clinical tests of lower body muscle function should therefore be interpreted cautiously if administered to unilateral LLP users. Future research to develop and assess the validity of clinically feasible methods for quantifying within and between limb strength deficits among unilateral LLP users is required.

### Several limitations should be considered when interpreting the results of the current study

Beyond amputation etiology, characteristics of the LLP user sample (e.g., age, amputation level, and perceived mobility) were largely consistent with those reported in large national studies of LLP users (i.e., n = 146–1568) [89–93]. While the results of this study may therefore generalize to the broader population of established unilateral non-dysvascular LLP users, they are limited to the characterization of *isometric* hip muscle function by *peak torque* at a single joint angle. Whether similar between limb differences are observed at different joint angles [94], during isokinetic muscle actions [95], and when other metrics of muscle function [45] including rate of torque development [96], steadiness [97], and endurance [98] are used to characterize hip muscle function remains to be determined. Data collection in the current study was lengthy and demanding. Multiple trials were performed to assess four hip muscle groups across the intact and residual limbs. The burden placed on study participants may have induced varying degrees of mental and/or physical fatigue, which may have affected study results. Mandatory rest periods and randomization of test conditions were implemented to minimize the systematic effect of participant fatigue and/or concentration. Aspects of sample heterogeneity may have influenced study results. While several sources of sample heterogeneity were managed through normalization (i.e., body size), statistical analysis (i.e., amputation level), and inclusion or matching criteria (i.e., age and gender), other sources of heterogeneity (i.e., cause of amputation, time since amputation, and amputation technique) were not. The potential for cause of and time since amputation to confound study results is limited, as most LLP user participants (i.e., 75%) had amputations of non-dysvascular etiology, and time since amputation does not appear to be related to muscle strength in LLP users [31, 99, 100]. Nonetheless, future research examining the influence of these amputation-related factors on muscle function, and specifically amputation technique [101], is warranted. As with all research that examines muscle function, the results of the current study are subject to the chosen data collection and analysis methods. The variation in data collection methods throughout the literature [22] limits comparisons between studies. Consequently, the extent to which the results and conclusion presented herein diverge from or confirm prior findings is difficult to ascertain. Further, the lack of consistent methodologies across studies presents a challenge to the aggregation of key findings, and the formation of consensus regarding muscle function in LLP users. Consideration for alternative normalization models that do not presume geometric similarity among LLP users [25, 26] (e.g., a gamma rather than power function model), and the exploration of additional anthropometric scaling variables is also recommended to identify the most appropriate and effective strength normalization procedures in unilateral LLP users. The development and dissemination of standardized methods for the collection, analysis, and reporting of strength-related outcomes in LLP users is therefore needed.

## Conclusion

In this study we found residual limb hip strength of unilateral LLP users, as estimated by maximum voluntary isometric peak torque, and normalized to BM × TL, to be significantly greater than that of their intact limb, and equivalent to that of age- and gender-matched controls. We propose that the observed pattern of between limb differences in hip muscle strength may be attributed to the elevated and prolonged activation of residual limb hip muscles during ambulatory activities, and only detected after having identified and adjusted for confounding anthropometric variables through appropriate scaling techniques. The findings of this study challenge long-held beliefs regarding patterns of hip strength among unilateral LLP users and suggest that physical therapy interventions may need to target the intact limb, not just the residual limb. Further research is warranted to confirm and expand upon the present findings, while also identifying modifiable factors associated with hip strength deficits in LLP users. When seeking to describe and explain between and within limb patterns of hip muscle function among unilateral LLP users, researchers should consider additional measures of muscle function (e.g., rate of torque development and steadiness), isokinetic muscle actions, as well as the concurrent collection of electromyographic, imaging, physical activity, and gait data.

## Supplementary Information

**Additional file 1**. Study data and data dictionary.

**Additional file 2**. The slope coefficients (i.e., β-values) and accompanying 95% confidence intervals of linear regressions performed on log-transformed (**A**) non-normalized and (**B**) normalized hip extensor, flexor, abductor, and adductor maximum voluntary isometric peak torque versus log-transformed product of body mass (BM) and thigh length (TL) (BM x TL) for the residual and intact limbs of unilateral lower limb prosthesis users, as well as age and gender match controls.

## Data Availability

The dataset(s) supporting the conclusions of this article is(are) included within the article (and its additional file(s)).

## Abbreviations

BM: Body mass
INT: Intact
LLP: Lower limb prosthesis
RL: Residual limb
S: Non-normalized strength
S_n_: Normalized strength
TL: Thigh length
