# Supplementary material for: After scaling to body size hip strength of the residual limb exceeds that of the intact limb among unilateral lower limb prosthesis users
Source: J Neuroeng Rehabil. 2023 Apr 25;20:50. doi: 10.1186/s12984-023-01166-z (PMC10131313; doi:10.1186/s12984-023-01166-z)
Supplement: Supplementary file 2 — Additional file 2. The slope coefficients (i.e., β-values) and accompanying 95% confidence intervals of linear regressions performed on log-transformed (A) non-normalized and (B) normalized hip extensor, flexor, abductor, and adductor maximum voluntary isometric peak torque versus log-transformed product of body mass (BM) and thigh length (TL) (BM x TL) for the residual and intact limbs of unilateral lower limb prosthesis users, as well as age and gender match controls. [file 12984_2023_1166_MOESM2_ESM.docx]

| Supplemental Material 1. The slope coefficients (i.e., β-values) and accompanying 95% confidence intervals of linear regressions performed on log-transformed (A) *non-normalized* and (B) *normalized* hip extensor, flexor, abductor, and adductor maximum voluntary isometric peak torque versus log-transformed product of body mass (BM) and thigh length (TL) (BM x TL) for the residual and intact limbs of unilateral lower limb prosthesis users, as well as age- and gender match controls. | | | | |
| --- | --- | --- | --- | --- |
| 1. Non-normalized | | | | |
|  | Hip extensors | Hip flexors | Hip abductors | Hip adductors |
| Residual limb | .326 (.011, .642)^(a)^ | .468 (.042, .894)^(a)^ | .354 (.036, .672)^(a)^ | .520 (.181, .859)^(a)^ |
| Intact limb | .607 (.242, .972)^(a)^ | .688 (.210, 1.17)^(b)^ | .727 (.285, 1.18)^(b)^ | .729 (.280, 1.18)^(b)^ |
| Control limb | .340 (.027, .652)^(a)^ | .390 (.010, .770)^(a)^ | .396 (.010, .784)^(a)^ | .504 (.212, .797)^(a)^ |
|  | | | | |
| 1. Normalized | | | | |
|  | Hip extensors | Hip flexors | Hip abductors | Hip adductors |
| Residual limb | .000 (-.315, .316)^(c)^ | .000(-.426, .426)^(c)^ | .000 (-.318, .318)^(c)^ | .000 (-.339, 339)^(c)^ |
| Intact limb | .000 (-.365, .365)^(c)^ | -.311 (-.790, .167)^(c)^ | -.271 (-.714, .171)^(c)^ | -.271 (-.719, .178)^(c)^ |
| Control limb | .000 (-.313, .312)^(c)^ | -.063 (-.450, .324)^(c)^ | .000 (-.388, .388)^(c)^ | .000 (-.292, .293)^(c)^ |
| β: slope coefficient; CI: confidence interval  (a): significant non-linear association between peak torque and anthropometric variable (CI between 0 and 1, p < .05)  (b): significant linear association between peak torque and anthropometric variable (CI includes 1 or greater but not 0, p < .05)  (c): no significant association between peak torque and anthropometric variable (CI includes 0 and not 1, p ≥ .05) | | | | |

For a detailed description of allometric scaling applied to the normalization of hip strength in unilateral lower limb prosthesis users, see Sawers and Fatone (2022). The following is a short summary: (A) The 95% confidence intervals around the slope coefficients (i.e., β-values) of the log-transformed regressions performed on non-normalized peak isometric hip torques versus the product of body mass and thigh length in the residual and control limbs fell between and excluded the values of 1.0 and zero. Interpretation of confidence intervals indicated that the slope of the log-log regression lines was significantly greater than zero, the association between peak hip torques and body mass x thigh length in the residual and control limbs was *non-linear*, and that normalizing isometric strength to body size was appropriate. In the intact limb of unilateral lower limb prosthesis users, the 95% confidence interval for peak hip extension torque had a similar *non-linear* association with body mass x thigh length (i.e., confidence interval values fell between and excluded 1.0 and zero). Peak hip flexion, abduction, and adduction torques in the intact limb however had a significant *linear* association with body-mass x thigh length, as indicated by 95% confidence intervals around the β-values including 1.0, but not zero (28, 48), again indicating that normalization to body size was required. For each significant *non-linear* association, the calculated empirical β-value was used as the scaling exponent to normalize peak torque (i.e., allometric scaling). For all significant *linear* associations, a theoretical β-value of 1.0 was used as the scaling exponent to normalize peak torque (i.e., ratio scaling) (28, 48). (B) Once normalized to body mass x thigh length, the 95% confidence intervals around the slopes of the log-transformed regressions between normalized peak hip torque and body mass x thigh length included the value of zero but not 1.0, indicating that the slopes of the normalized log-log regressions were *not* significantly greater than zero in all four muscle groups, across all three limbs (28, 48). Normalization of peak isometric hip torque to body mass x thigh length effectively reduced the dependency of peak hip torque on body size, creating body size independent measures of hip strength that are comparable between people and legs that differ in size (28, 48).
